# Identification of an elusive *SERPING1* deletion in a family with hereditary angioedema type I utilizing soft clipping

**DOI:** 10.3389/falgy.2025.1565283

**Published:** 2025-04-17

**Authors:** Keith Wetherby, Joseph Chiao, Emily Faulkner, Yongjian Guo, Shaobin Hou, J. Joanna Yu, Jinguo Chen, Lili Wan, H. Henry Li

**Affiliations:** ^1^ Virant Diagnostics, Inc., Wheaton, MD, United States; ^2^ ScitechLink, LLC, Rockville, MD, United States; ^3^ Institute for Asthma and Allergy, Chevy Chase, MD, United States

**Keywords:** SERPING1, hereditary angioedema (HAE), multiplex ligation-dependent probe amplification (MLPA), c1-inhibitor (C1INH), soft clipping, next generation sequencing (NGS)

## Abstract

**Background:**

Hereditary angioedema (HAE) is an autosomal dominant genetic disorder caused by mutations in the C1 esterase inhibitor gene, SERPING1, leading to overproduction of bradykinin and debilitating swelling attacks. Variants in the *SERPING1* gene are typically detected in a clinical setting by DNA sequencing or multiplex ligation-dependent probe amplification (MLPA), with over 893 total variants identified. Approximately 5% of patients with C1-esterase inhibitor deficiencies do not have detectable *SERPING1* pathogenic variants. We further investigated a family with laboratory-confirmed HAE type I despite previous negative genetic test results for *SERPING1* mutations.

**Methods:**

We consented and collected whole blood samples from three family members with clinical diagnoses of HAE. The samples underwent genomic DNA extraction and evaluation for purity prior to sequencing. The DNA samples were processed through a semi-automated whole exome library prep pipeline and sequenced. *SERPING1* MLPA was performed to assess exon-level copy number variation (CNV) for exons 1 through 8. Additionally, we incorporated a well-established bioinformatics technique called soft clipping into our variant analysis pipeline to detect structural variants.

**Results:**

Clinical variant analysis revealed two common benign variants of *SERPING1* in the proband. NGS and MLPA did not detect any *SERPING1* pathogenic variants or genomic rearrangements, but additional structural variant analysis identified a high rate of soft clipping in exon 6 of the *SERPING1* gene. Sanger sequencing of exon 6 revealed a heterozygous 56-base-pair deletion [NC_000011.10: g.57606508-57606563del, NM_000062(*SERPING1*): c.990_1029 + 16del] spanning the 3’ exon-intron boundary in all three subjects.

**Summary:**

Without additional techniques following NGS and MLPA, such as a soft clipping analysis method, many difficult-to-detect large insertions and deletions may go undetected. We propose that a systematic approach to undetected HAE-causing mutation analysis, incorporating soft clipping as part of an overall strategy, would be more effective in identifying a small percentage of causal variants in approximately 5% of C1-esterase inhibitor HAE cases where no mutation is found by standard laboratory procedures, especially when there are high clinical suspicions of a familiar disorder.

## Introduction

Hereditary angioedema (HAE) is a rare, life-threatening autosomal dominant genetic disease, most commonly caused by mutations in the *SERPING1* gene on chromosome 11q. These mutations disrupt the regulation of the kallikrein-kinin system (KKS), resulting in the overproduction of bradykinin. Without treatment, angioedema episodes can be severely debilitating and may result in fatal laryngeal asphyxiation. To date, more than 893 *SERPING1* variants have been identified, primarily through genetic techniques such as next-generation sequencing (NGS) and multiplex ligation-dependent probe amplification (MLPA) (1). However, clinicians must be aware of the limitations of these methods, especially when negative genetic test results conflict with clear clinical presentations of HAE. This highlights the importance of thorough clinical evaluation and a general understanding of genetic testing methods to ensure accurate diagnosis and effective patient management.

The *SERPING1* gene contains 17 Alu elements, making it susceptible to non-homologous recombination events and prone to large deletions, insertions, and duplications (2, 3). Alu-repeat-mediated variations account for approximately 10% of all HAE cases (4). Due to technical limitations, conventional NGS short-read sequencing may miss small and large heterozygous rearrangements (5). Therefore, other molecular techniques, such as MLPA, are needed to investigate these events.

Herein, we describe three family members with HAE type I who test negative for pathogenic variants and large genomic rearrangements in *SERPING1*, as determined by NGS and MLPA, respectively. Using a systematic approach to HAE-causing mutation analysis that incorporates soft clipping, we identified a novel heterozygous disease-causing deletion in *SERPING1*, responsible for the C1 inhibitor deficiency in affected family members.

## Materials and methods

### Ethics declarations

All subjects analyzed in this study gave written informed consent before participation. The study protocol was approved by the Salus IRB (IRB protocol number: Virant-A0001).

### HAE subtypes

Mutations in the *SERPING1* gene cause two types of HAE. HAE-C1INH type I is caused by a quantitative C1 esterase inhibitor protein deficiency mainly due to truncating mutations. HAE-C1INH type II is caused by the reduced function of the C1 esterase inhibitor protein mainly resulting from missense or in-frame variants at or near the reactive site P1-P1’ (Arg466-Tyr467) on the exposed reactive mobile loop in exon 8 (6). HAE type I and type II affect about 1 in 50,000 people, with 85% of HAE patients representing type I (7, 8). NGS is typically used for molecular confirmation of HAE type I and type II in medical genomics studies.

### Patient demographics

The clinical history of recurrent chronic angioedema and diagnostic test results for three family members (35-year-old female proband, her 8-year-old son, and her 62-year-old mother) were suggestive of HAE type I, characterized by low levels of complement C4, C1 esterase inhibitor antigen, and C1 esterase inhibitor function. Previous genetic testing of *SERPING1* for each family member, conducted at another genetics laboratory, did not identify any mutations.

### Blood collection + genomic DNA extraction

Whole blood was collected from the patients in BD vacutainer K2 EDTA tubes (Beckton Dickenson) and then stored at 4°C until DNA extraction. Genomic DNA was extracted from the patient's blood on the Qiagen EZ2 Connect MDx using the EZ1&2 DNA Blood 350ul kit (Qiagen) following the manufacturer's instructions. The DNA concentration and purity were evaluated using the Qiaxpert system (Qiagen).

### Next generation sequencing (NGS)

Genomic DNA samples were processed through a semi-automated library prep pipeline (Agilent SureSelect Human All Exon V8, Agilent Magnis) and sequenced using Illumina technology. Reads were aligned and mapped to GRCh38 using Agilent Reporter v1.3.1 software. Variant filtering was performed using cloud-based commercial software (Agilent Interpret v5.4.2). The impact of variants was evaluated using commercial (Alamut Visual Plus, Sophia Genetics) and publicly available computational tools. Read alignment was visualized using Integrative Genomics Viewer (Broad Institute). Variants were classified according to ACMG guidelines (9).

### MLPA

**Figure 1:**
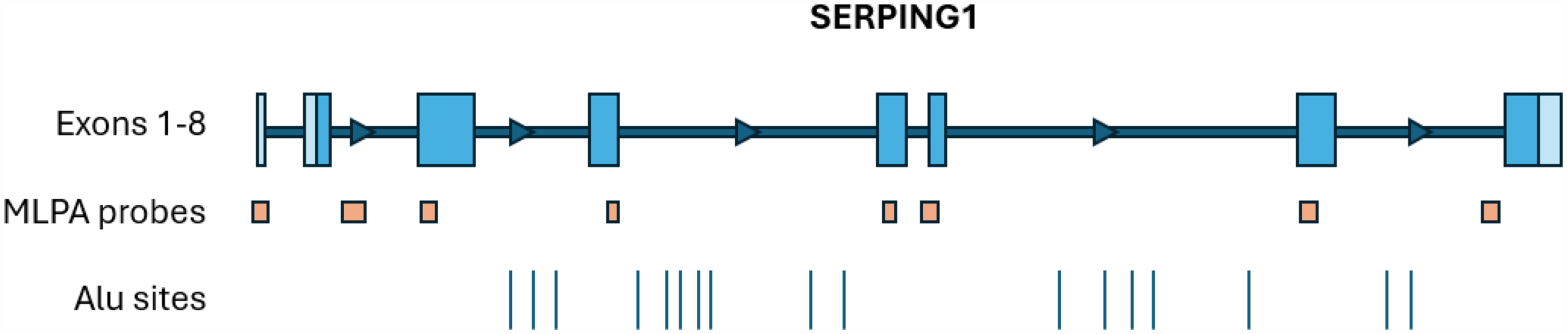
The approximate locations of *SERPING1* exons, MLPA probes (MRC holland, probemix P243 *SERPING1-F12*), and Alu sites (2). The MLPA probe locations are as follows: ex1 probe covers the entire exon, ex2 probe is downstream of exon 2, ex3 probe is at the 5’ end of exon 3, ex4 probe is at the 3’ end of exon 4, ex5 probe is within exon 5, ex6 probe is on the 5’ intron-exon boundary, ex7 probe is at the 5’ end of the exon, and the ex8 probe is upstream of exon 8.

Exon level copy number variation (CNV) for exons 1 through 8 in *SERPING1* was performed using Salsa MLPA Probemix P243 (MRC Holland) following the manufacturer's instructions. The probe mix kit consists of 11 control probes, 8 *SERPING1* probes, 13 FXII probes and 1 APLNR probe, a gene upstream of *SERPING1*. Four DNA controls, including 3 healthy reference samples and one positive sample for a *SERPING1* exon 4 deletion, were run with patient samples on an ABI 3500xl Genetic Analyzer (Applied Biosystems). Data analysis was performed using Coffalyser software v.240129.1959. The location of MLPA probes in relation to each *SERPING1* exon is shown in Figure 1.

### Sanger sequencing

Custom primers (forward, 5’-TCGGATCTCAATGTCCCTGC-3’ and reverse, 5’-TTGAGAATCCTGTTTCCAGCCT-3’) (Integrated DNA Technologies) were used to amplify a 459-base-pair product for *SERPING1* exon 6 using the following PCR conditions: initial denaturation 98°C for 30 s followed by 35 cycles of denaturation at 98°C for 10 s, annealing at 60°C for 10 s and extension at 72°C for 90 s. The PCR product was cleaned up using Exo-SAP (Applied Biosystems) following the manufacturer's instructions. Bidirectional Sanger sequencing using Big Dye Terminator v3.1 (Applied Biosystems) and subsequent clean-up using Xterminator beads (Applied Biosystems) were performed following the manufacturer's instructions. Capillary electrophoresis was performed on a 3500xl Genetic Analyzer (Applied Biosystems). Analysis of the sequencing data was performed using Genecodes Sequencher (RRID:SCR_001528) version 5.4.6.

### Bioinformatics tools

We implemented a well-established bioinformatics technique called soft clipping into our analysis pipeline, using BWA, BEDTOOLS, and BEDMAP, to detect potential genomic rearrangements in the *SERPING1* gene (10–12). Soft clipping regions in NGS sequence alignment represent bases that do not align with the reference sequence without removing them from the read data. The clipping percentage is calculated by dividing the number of reads with soft clipping bases by the total number of reads. The clipping percentage for a given exon is then compared to other samples. An increase in percentage compared to the healthy samples suggests further investigation is needed. Low-quality MAPQ scores were filtered out of the analysis.

## Results

### NGS

Variant analysis for *SERPING1* revealed two common benign variants (Table 1) in the proband but not in the mother or the son. Due to their high frequency in population databases (gnomAD, Broad Institute) and our lab, these variants were not given further consideration.

**Table 1:** Benign variants in *SERPING1***.**

Benign variants in *SERPING1*					
Proband					
Sample	Gene	Transcript	Coding	Protein	gnomAD
0002-HAE-001	*SERPING1*	NM_000062	c.1030-20A>G	n/a	65%
0002-HAE-001	*SERPING1*	NM_000062	c.1438G>A	p.Val480Met	25%
Mother and Son					
No variants detected in *SERPING1*					

This table indicates that two common benign *SERPING1* variants were found in the proband, but not in the mother or son.

### MLPA

Due to the presence of 17 Alu elements, MLPA is used as part of the systematic analysis of *SERPING1* when no pathogenic variant is found during DNA sequencing. One positive control sample with a heterozygous exon 4 deletion in *SERPING1* and three healthy reference samples were tested along with the proband and mother. The proband and mother were negative for copy number variations in exons 1–8. Therefore, we did not test the son. Negative MLPA results do not completely rule out structural variants >50 bp (13). Deletions or insertions occurring outside the MLPA probe locations will go undetected.

### Soft clipping analysis

**Figure 2:**
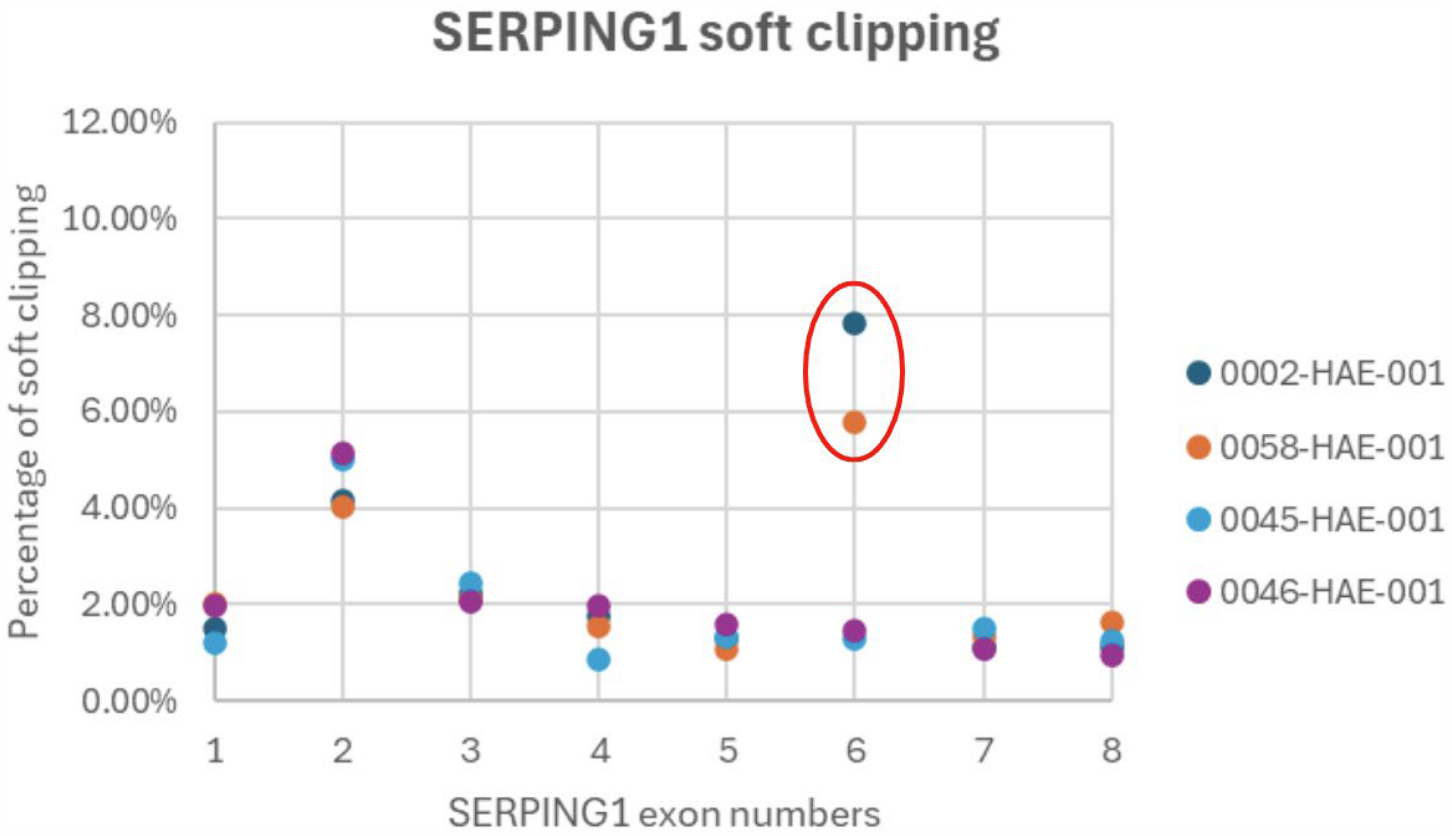
A high rate of soft clipping in *SERPING1* exon 6. Soft clipping percentages graphed per patient for *SERPING1* exons 1-8. Outliers are circled red and suggest further investigation.

All three family members have low levels of C1-INH indicating a *SERPING1* mutation, but no pathogenic mutations were found through NGS or MLPA. Soft clipping is a bioinformatics technique used in sequence alignment to mark bases that do not align with the reference sequence without removing them from the read data. We processed the NGS bam files for the proband, mother (son not tested) and two additional controls through our soft clipping bioinformatics workflow to investigate the *SERPING1* gene for difficult-to-detect insertions and deletions (14). Of the two control samples, 0046 had a known heterozygous *SERPING1* exon 4 deletion, and 0045 had a missense *SERPING1* mutation in exon 3. A high rate of soft clipping was detected in *SERPING1* exon 6 in this HAE family as shown in Table 2; Figure 2.

**Table 2:** A high rate of soft clipping in *SERPING1* exon 6.

Exon	0002-HAE-001	0058-HAE-001	0045-HAE-001	0046-HAE-001
1	1.52%	2.01%	1.21%	1.99%
2	4.14%	4.04%	5.01%	5.12%
3	2.25%	2.11%	2.45%	2.06%
4	1.77%	1.53%	0.86%	1.96%
5	1.29%	1.06%	1.35%	1.57%
6	**7**.**84%**	**5**.**79%**	1.28%	1.45%
7	1.11%	1.33%	1.49%	1.09%
8	1.11%	1.65%	1.26%	0.95%

This table presents the percentage of soft clipped reads for each patient sample across *SERPING1* exons 1–8. The bolded percentages for exon 6 indicate an increase in soft clipped reads for both the proband and mother compared to control samples. Control sample 0046, which carries a heterozygous deletion of exon 4, does not show an increase in the percentage of soft clipped reads. Exon 2 has a higher percentage of soft clipped reads for all samples due to the high GC content of the sequence (∼70%).

### Sanger sequencing

**Figure 3:**
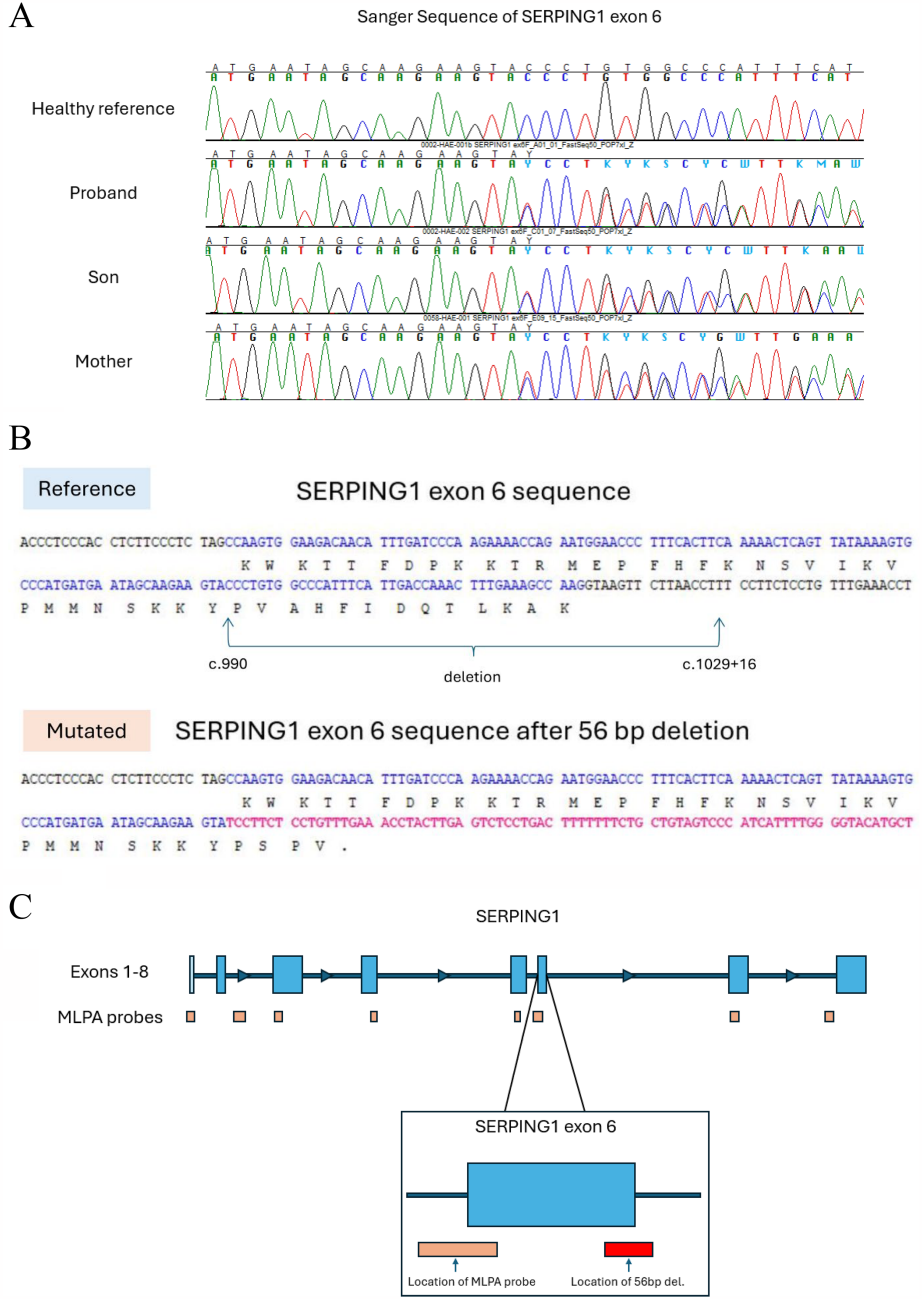
Sanger sequencing of exon 6 revealed a heterozygous 56-base-pair deletion. **(A)** Sanger electropherogram showing *SERPING1* exon 6 for the three family members and a healthy control. **(B)** The reference nucleotide sequence of exon 6 (top) and the deleted sequence found in the HAE patients (bottom). The intron sequence is shown in black; the exon sequence is shown in blue with the corresponding amino acid code shown below; the new sequence after the deletion is shown in red. A new stop codon is introduced shortening the transcript to 335 total amino acids. **(C)** The approximate location of the MLPA probe and the 56-base-pair deletion show that a large structural variant >50 bp can be missed.

We performed Sanger sequencing for *SERPING1* exon 6 for all three family members based on an increase of soft clipped reads compared to controls. A novel disease-causing heterozygous 56-base-pair deletion, [NM_000062 (*SERPING1*): c.990_1029+16del], spanning the 3’ exon-intron boundary was subsequently identified for all three family members. The deletion is predicted to cause a frameshift at coding position 332 resulting in the addition of 3 spurious amino acids before a premature translational stop codon (p.Val332Serfs*3) as shown in Table 3; Figures 3A–C.

**Table 3:** Pathogenic *SERPING1* mutations**.**

Pathogenic *SERPING1* Mutations			
Proband, Mother, Son			
Gene	Transcript	Coding	Genomic
*SERPING1*	NM_000062	c.990_1029+16del	NC_000011.10:g.57606508_57606563del

This table indicates the presence of a heterozygous *SERPING1* pathogenic variant in the proband, mother, and son.

## Discussion

We performed NGS sequencing and MLPA for three family members with chronic recurrent angioedema and abnormal C4 and C1-INH screening tests but found no obvious *SERPING1* mutation. Prior analysis by another genetics laboratory also failed to detect *SERPING1* mutations. Due to the high clinical suspicion, the allergist requested further investigations by our laboratory to identify a potential genetic association in this family. In response, we developed a soft clipping bioinformatics workflow to analyze NGS data for difficult-to-detect insertions and deletions. Results indicated a high percentage of soft clipping in *SERPING1* exon 6 and Sanger sequencing identified a novel 56-base-pair deletion. The *SERPING1* c.990_1029+16del variant likely creates a new premature stop codon, subjecting the transcript to nonsense-mediated mRNA decay (NMD).

The NMD quality control system ensures that potentially toxic polypeptide fragments do not accumulate while simultaneously coping with possible translational logjams that might arise from the inherent inefficiency of premature translation termination (15). The early stop codon and NMD explain the C4 and C1-INH deficiency in the three affected family members diagnosed with HAE type I.

We believe that a systematic approach to undetected HAE analysis, using soft clipping as part of an overall strategy, would be effective in determining a small percentage of causal variants in approximately 5% of HAE-C1INH cases where no mutation is found by standard clinical NGS procedures (16–18). Vatsiou 2020 used splice analysis to discover deep intronic cryptic splice sites. Splice site analysis is also an effective tool for finding potential pathogenic variants and SpliceAI (Broad Institute) is included in our overall strategy for undetected HAE analysis. SpliceAI is a deep neural network that models mRNA splicing and is used to predict cryptic splice sites in noncoding genomic sequences (19). Deep intronic variants are seldom covered in routine HAE clinical sequencing, making it easy for these variants to go undetected.

Identification of genetic variants, particularly those involving *SERPING1*, is critical for several reasons. Confirming a genetic mutation supports the patient's symptoms and clinical diagnosis of HAE, particularly in patients with overlapping or atypical symptoms. Determining the underlying genetic cause can guide targeted therapeutic interventions, ensuring the patient receives the most effective treatment for their disease subtype. In addition to receiving personalized treatment, it documents proof of the patient's disorder for insurance coverage of potentially expensive and chronic therapies for HAE. A clinical genetic diagnosis also enables family screening and early identification of at-risk relatives, allowing for possible life-saving preventative measures or early treatment interventions.

Soft clipping bioinformatics analysis reduces the need for exploratory genetic testing and facilitates molecular diagnosis through a more targeted approach. This method enables high-throughput screening and provides a more comprehensive analysis of NGS data compared to visual inspection of sequencing reads alone. Retrospective analysis of previously sequenced samples from undetected HAE patients can be performed, conserving valuable time and resources that would otherwise be allocated to exploratory sequencing efforts.

The use of soft clipping in genetic testing represents an important step in addressing diagnostic challenges in rare diseases like HAE. This approach marks areas for additional review that standard clinical analysis may miss, providing clinicians and patients with a more comprehensive diagnostic toolset. These findings can inform future research efforts and contribute to the development of novel therapeutic approaches.

Clinicians and genetic testing laboratories should collaborate closely to ensure systematic and thorough evaluation of suspected HAE cases, particularly in those with strong clinical suspicion and negative initial genetic testing results. The limitations of genetic methodologies should be acknowledged and discussed. It should be noted that NGS sequencing only detects pathogenic mutations in approximately 80%–85% of HAE cases, including single nucleotide variations such as missense, nonsense, and small insertions and deletions < 15 base pairs. Standard variant analysis commonly covers the exon and flanking sequence, so deep intronic splice variants, if present, are often excluded. The incorporation of MLPA to detect large deletions, insertions, and duplications, accounts for another 10% of HAE cases. Nevertheless, similar to the case of this reported family, large deletions may sometimes go undetected. The technical limitations of NGS and MLPA in detecting structural variants can be overcome with long-range sequencers, such as Pacific Biosciences’ single-molecule real-time sequencing (PacBIO) and Oxford Nanopore Technologies nanopore sequencing (ONT). However, these long-read sequencing technologies present their own limitations in base calling accuracy, with a single-pass accuracy of 85%–87% (20). Although Sanger sequencing is still considered the gold standard in sequencing and helped us detect this family's mutation, its low throughput limitations have led to it being largely replaced by high-throughput, cost-effective NGS in most clinical genetics laboratories.

To enhance the diagnostic accuracy of hereditary angioedema (HAE) caused by *SERPING1* mutations, we recommend adhering to the general testing algorithm outlined by the World Allergy Organization and the European Academy of Allergy and Clinical Immunology (21). Clinicians ordering genetic tests should be informed about the laboratory's methodology and sequencing technology, particularly for rare diseases such as HAE, where multiple types of genetic variations exist within *SERPING1*. Most genetic laboratories have transitioned from Sanger sequencing—traditionally the preferred method for *SERPING1* analysis—to next-generation sequencing (NGS) for greater efficiency. However, when NGS and multiplex ligation-dependent probe amplification (MLPA) yield negative results, soft clipping analysis should be considered. This analysis must be performed alongside other samples in the same NGS run, as variations in sequencing runs, laboratory pipelines, and instruments can introduce inconsistencies that affect reliability. Selecting a genetics laboratory with the capability to conduct additional NGS bioinformatics analyses is essential for improving mutation detection in *SERPING1* and optimizing HAE diagnosis.

The Leiden Open Variation Database (LOVD) contains five *SERPING1* deletion variants greater than 50 bases but less than an exon, as of the date of this publication. These variants (*SERPING1* NM_000062: c.138_207del69, c.250_414del165, c.726_777del51, c.1391_1445del55, and c.*101_*254del153) were all identified by Sanger sequencing (16, 22–25). Generally, NGS *SERPING1* studies report variations of small frame shifts and large gene rearrangements. To emphasize the importance of NGS limitations, the creation of an intermediate frame shift category, defined as greater than 50 bp and less than an exon, should be considered. The limitations of any laboratory's sequencing technology should be clearly stated on genetic test reports, as required by clinical regulatory agencies. It is suggested that the undetected 5% of HAE mutations may be found in intronic or untranslated regions of the gene. While we do not refute this stance, we want to expand this suggestion to cover technological limitations as well.

As commercial genetic laboratories increasingly adopt NGS as their primary method, replacing Sanger sequencing to improve overall efficiency, the integration of more techniques—such as soft clipping and splice site analysis—should be prioritized to further close diagnostic gaps and optimize patient care, despite the additional costs. Clinicians ordering genetic tests must be well-informed and vigilant, particularly when familial clinical symptoms suggest a genetic etiology that is not evident in negative test results. Enhanced awareness of these limitations can prompt reanalysis or alternative testing strategies. Furthermore, interdisciplinary collaboration to integrate these advanced techniques into routine NGS workflows holds the potential to improve diagnostic yields, facilitate timely access to effective treatments, and advance equitable healthcare for patients with rare and complex diseases.

## Data Availability

Public database of variant discussed in the article was sent to ClinVar and acknowledged on March 12, 2025, clinvar@ncbi.nlm.nih.gov, File name(s): Submission ID: SUB15167532, Organization ID: 509915.
